# Ramadan during pregnancy and the role of dietary intake for neonatal health in Kaduna, Northwestern Nigeria: a cross-sectional study

**DOI:** 10.1186/s12884-025-07158-2

**Published:** 2025-01-23

**Authors:** Musa Abubakar Kana, Bilkisu Usman, Amina Mohammed-Durosinlorun, Jennifer Ahmed, Hassana Habiba Abubakar, Yakub Abdulmalik, Fabienne Pradella

**Affiliations:** 1 Chair of Statistics and Econometrics, Faculty of Law, Management and Economics, Johannes Gutenberg-University, Mainz, Germany; 2 Department of Community Medicine, College of Medicine, Kaduna State University, Kaduna, Kaduna State Nigeria; 3 Kaduna Infant Development (KID) Birth Cohort Study Project, Center for Research in Developmental and Life Course Epidemiology (CREDLE), Kaduna, Nigeria; 4 Department of Obstetrics and Gynaecology, College of Medicine, Kaduna State University, Kaduna, Kaduna State Nigeria; 5 Heidelberg Institute of Global Health, Heidelberg University, Heidelberg, Germany; 6 Division of Primary Care and Population Health, Department of Medicine, Stanford University, Stanford, CA USA

**Keywords:** Ramadan, Pregnancy, Intermittent fasting, Birth Weight, Gestational age, Nigeria

## Abstract

**Background:**

Ramadan during pregnancy is associated with adverse offspring health outcomes. Recent evidence from Europe indicates that maternal diet during non-fasting hours might alleviate these effects. This study describes fasting, diet, and sleep habits among pregnant Muslims in Kaduna, Nigeria, and assesses impacts on neonatal health in this setting.

**Methods:**

Between July 2023 and February 2024, we conducted 1814 interviews with women whose pregnancy overlapped with Ramadan 2023 in Kaduna, northwestern Nigeria (cross-sectional study). We used a structured questionnaire to collect data on fasting, diet and sleep during Ramadan, as well as maternal socio-demographic characteristics. In the analyses of birth outcomes, 1370 mother-newborn pairs were included. We performed multivariate regression analyses to evaluate associations between fasting and birth weight and gestational duration, as well as the interaction of fasting with maternal dietary intake and sleep patterns.

**Results:**

More than 80% of the study participants fasted during pregnancy. Women who fasted had infants with lower birth weights than non-fasting participants (-90.38 g, 95% CI: -173.64 g to -6.12 g). No independent associations were detected between reduced sleep and food intake, and birth weight. Fasting was not associated with gestational duration. Negative effects of fasting on birth weight were consistently concentrated among participants who reduced their dietary or fluid intake during Ramadan.

**Conclusions:**

Dietary intake during non-fasting hours might mitigate potential adverse effects of Ramadan fasting on birth weight. With more than 25% of the global population adhering to Islam, this study highlights the need for additional research on Ramadan during pregnancy across different settings.

**Supplementary Information:**

The online version contains supplementary material available at 10.1186/s12884-025-07158-2.

## Background

Suboptimal maternal nutrition is associated with adverse offspring health, education and labor market outcomes over the life course [1, 2]. While the earlier literature focused on extreme nutritional deprivation during pregnancy such as historical famines [3, 4], more recent studies investigated milder and time-constrained nutritional shocks during pregnancy, which are also more common. One prevalent form of milder nutritional deprivation during pregnancy is intermittent fasting, such as breakfast skipping [5, 6]. Intermittent fasting is also observed in the context of cultural and religious fasting, followed by adherents of various faiths [7]. This includes daytime intermittent fasting during Ramadan, a period of important spiritual reflection for Muslims.

Ramadan fasting, a core practice of Islam, overlaps with most pregnancies among the approximately 1.9 billion Muslims worldwide. Ramadan lasts 29–30 days and is characterized by fasting during daylight hours and unrestricted food and drink intake at night. Pregnant women are exempt from fasting if they have concerns for their own or their baby’s health. A substantial fraction of pregnant Muslims in Asia and Europe decide to fast. Studies in the Netherlands (54%) and the UK (43%) show lower fasting rates than in Pakistan (88%) or rural Bangladesh (99%) [8–11]. The only available evidence on Ramadan during pregnancy in Africa is a recent study on Morocco, which documents a fasting rate of 89%, however only reporting on the third pregnancy trimester [12]. Ramadan during pregnancy has been associated with various adverse offspring outcomes, including childhood growth faltering, infant mortality, and chronic conditions in adulthood [13–16]. In contrast, for healthy non-pregnant adults, Ramadan fasting has been shown to improve lipid profile, and overall metabolic and cardiovascular health [17–22].

In this study, we describe the determinants of fasting among pregnant Muslims in Kaduna, Northwestern Nigeria, using detailed survey data on a cross-section of 1370 Muslims whose pregnancies overlapped with Ramadan 2023. Additionally, we examine associations between fasting and neonatal health, linking survey data with hospital-based information.

We focused on whether altered dietary intake and sleep patterns during Ramadan influence the effects of fasting on birth outcomes. Beyond fasting, Ramadan involves changes to the dietary composition. Dietary intake during Ramadan is characterized by traditional meals such as during the breaking of the fast at sunset, which vary across Muslim communities around the globe [23, 24]. Furthermore, sleep rhythms and food and drink intake are adapted because its preparation and intake are shifted to night hours [25, 26]. While behaviors beyond fasting have been hypothesized to modify the health effects of fasting during pregnancy, most previous literature did not have the necessary data to pursue this investigation. To date, the only study linking fasting, dietary intake, sleep and birth outcomes found that adverse neonatal health outcomes are identified only among women who reduced their dietary intake, particularly in terms of fatty food, during Ramadan in Germany [27]. However, it remains unclear if this finding also applies to other settings.

Research on how diet and sleep during Ramadan, beyond and in combination with fasting, is associated with offspring health is highly relevant for many pregnant Muslims who consider fasting during Ramadan, as well as for their health counsellors.

## Methods

### Data sources and study population

We conducted a cross-sectional study among Muslim mothers in Kaduna, Northwestern Nigeria. Kaduna is currently the 4th most populous city in Nigeria, comprising 1.6 million inhabitants with socially heterogenous backgrounds and a sizable proportion of Muslims. The target population of this study consisted of all Muslim women delivering singleton babies in the selected hospitals in Kaduna, whose pregnancy overlapped with Ramadan 2023 (March 23rd – April 21st, 2023). Multiple births were excluded due to their higher risks for preterm birth and low birth weight.

The study was implemented from 1 July 2023 to 26 February 2024 at the obstetric ward and immunization clinic of Yusuf Dantsoho Memorial Hospital and the immunization unit of the Children’s Clinic, Tudun Wada. These two hospitals have high attendance for antenatal care, facility deliveries and newborn immunization in the Kaduna South Local Government Administrative area. Kaduna South represents the most populous among four administrative divisions of the city of Kaduna.

The participants were recruited and administered the study questionnaire at the study sites. The recruitment of participants at both obstetric wards and immunization clinics was pivotal in the setting, since only 32.4% of deliveries in Kaduna take place in hospitals, while 78.3% of newborns receive the first Bacillus Calmette-Guérin (BCG) vaccination and a growth monitoring within their first week of life [28]. The participant recruitment in obstetric wards and immunization clinics ensured that both mothers that delivered in the health facility or at home were included in the sampling frame of the study.

We used a structured questionnaire to collect information on fasting, diet and sleep during Ramadan, as well as maternal socio-demographic characteristics (Supplementary File 1). The questionnaire was validated in a previous study [27] and further adapted to the local context for this project. All interviews were conducted by trained female enumerators with tertiary education in nursing-midwifery or nutritional sciences. The data was collected using *KoboToolbox* software on a tablet computer. Only female interviewers were employed to comply with the cultural expectations in the setting. The interviews were held in English (official language in Nigeria) or Hausa (most widely spoken language in Kaduna and most of Northern Nigeria), depending on the participant’s language preferences. All interviewers were fluent in both languages. The questionnaire was translated and back translated to Hausa to ensure that the original context was maintained. Subsequently, the survey data was linked to maternal demographic and birth data from obstetric ward and/or immunization clinic.

The study conformed to the principles embodied in the Declaration of Helsinki. All participants provided informed consent. The participants were assured that the study was anonymous, and their privacy and confidentiality protected by the removal of identifying information during all the stages of data management, analysis, and dissemination.

**Fig. 1:**
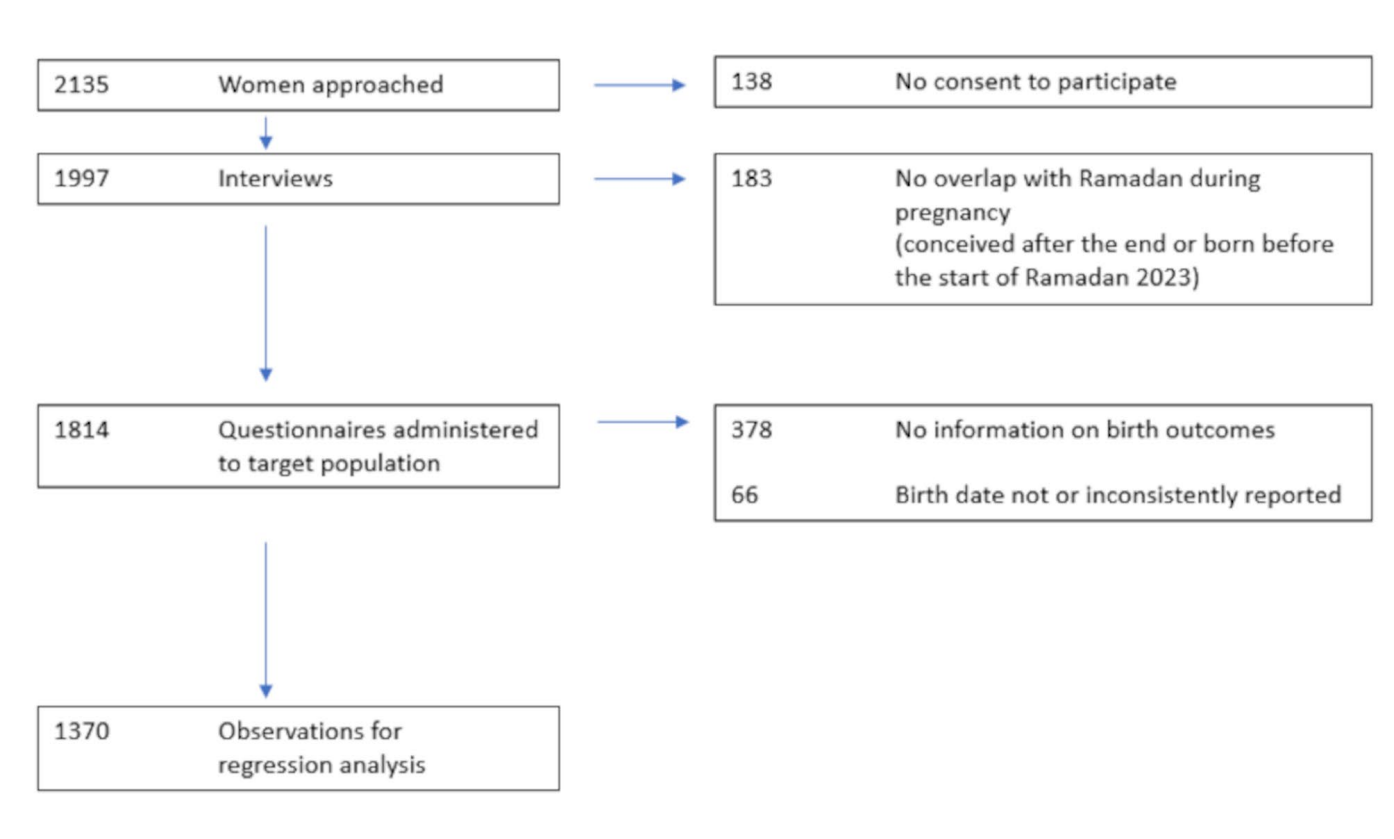
STROBE flow diagram of the sample recruitment

We conducted 1814 interviews with women whose pregnancy overlapped with Ramadan 2023. In the analyses of birth outcomes, 1370 mother-newborn pairs were included (Fig. 1). Given the high participation rate among the women approached, the sample can be considered representative of Muslim women delivering in Kaduna South Local Government Administrative Area who visited one of the two study sites for facility delivery or newborn immunization, and whose birth information was recorded.

We excluded observations with inconsistent reports on the birth date from regression analyses, since the Ramadan-pregnancy overlap was calculated based on date of birth. Only observations with at least one neonatal health outcome (birth weight or gestational duration) were included in the regression analyses.

### Exposure: fasting, diet and sleep during Ramadan

The main exposure was maternal fasting during pregnancy. Babies were considered exposed if they were born to mothers who observed fasting for at least three days during their pregnancy. This definition establishes an exposure risk threshold to assess associations between regular fasting and offspring health. Women who fasted for a day or two were not considered to be fasting regularly: these participants indicated that they had tried fasting, but realized it was too difficult or for other personal reasons abandoned fasting immediately.

The additional exposures we evaluated were maternal diet and sleep during Ramadan. Beyond fasting, adjustments to Ramadan include the intake of food early in the morning and late at night and changes in nutrient intake [26, 29]. We asked women about changes in the total amount of food consumed, and more specifically patterns in simple sugar or starchy food, fatty foods, fruit and fluid intake. We used the month before Ramadan as the reference point for the study participants to compare their intake patterns during Ramadan with the month before Ramadan. We defined participants as having reduced the intake in the respective category if they reported a reduced intake as compared to the month prior to Ramadan. While information on general food intake was collected for both fasting and non-fasting women, information on specific food categories were only collected among fasting women.

Participants were considered to have slept less if they reported going to bed later or getting up earlier (or both) during Ramadan compared to the month before, while not napping more during the day. Research on maternal sleep and birth outcomes remains scarce, but the available evidence suggests that lack of maternal sleep or poor maternal sleep quality may be associated with adverse birth outcomes [30]. Information about sleep was collected for all women, independent of whether they fasted or not.

### Outcomes

The main outcome was birth weight (in grams), which has been shown to have predictive value for short- and long-term health, education and labor market outcomes [31–35]. It was recorded by trained nurses and midwives on the child health (“immunization and growth monitoring”) card. Measurements of the neonates were made without clothing using a digital scale either at birth in the health facility or within 48 h, during the BCG immunization visit, for home births. For analyses on birth weight, the sample was restricted to term births (≥ 37 to ≤ 42 weeks of gestation) in order to avoid collider bias [36].

Gestational duration (in weeks) was considered as secondary outcome. Prematurity is a main cause of death among newborns and under 5-year-olds. It is also predictive of long-term health effects such as hearing complications [37, 38]. Gestational duration (in days) was calculated based on self-reported last menstrual period (LMP) or, if LMP was not available, the deviation of the actual birth date from the calculated due date.

Birth outcomes were linked to the survey data upon consent of the study participants.

### Covariates

We adjusted for educational level, employment status, parity, infant sex, gestational age at birth and gestational age at birth squared, maternal length of stay in Kaduna (less or more than 5 years) and awareness of the pregnancy during Ramadan. We also adjusted for the trimester of pregnancy during which Ramadan occurred. If the overlap between a pregnancy and Ramadan 2023 spanned two pregnancy trimesters, we assigned the observation to the trimester with the greater number of days of overlap.

### Statistical analyses

First, we performed descriptive statistics on the sample. This included descriptive statistics on the reasons for and against fasting as well as comparisons of fasting and non-fasting pregnant women regarding their sociodemographic characteristics and behavioral adjustments to Ramadan.

Second, we used multivariate regression analyses to evaluate associations between fasting and birth weight. The study followed a complete case analysis approach, so that only observations with information on all covariates were included in each analysis.

Third, to investigate the role of behavioral adjustments to Ramadan, we additionally adjusted for decreased overall dietary intake and sleep during Ramadan. This model estimated whether other adaptations to Ramadan were associated with birth outcomes independent of the fasting decision.

Fourth, to explore whether the effects of fasting varied by dietary intake and sleep during Ramadan, we interacted the effect of fasting with the respective behavioral category.

This study is reported according to the Strengthening the Reporting of Observational Studies in Epidemiology guidelines [39]. The threshold of statistical significance was set at 0.05 and all analyses were conducted using *Stata 17.0 SE*.

### Sensitivity analyses

In a sensitivity analysis for the analysis on birth weight, we included all births, irrespective of gestational age at birth. Moreover, the stability of results was tested against the exclusion of single covariates. Finally, we included maternal age at birth in the models to test the robustness of our results. Although this variable was available for only about half of the sample, this sensitivity analysis is crucial because maternal age might be associated with both the maternal fasting decision as well as birth outcomes.

## Results

### Descriptives

Ramadan fasting is prevalent among pregnant Muslims in Kaduna with more than 80% of the study participants fasting during pregnancy (Table 1). Fasting women were more likely to be primipara, and more often sought information about Ramadan during pregnancy. Moreover, fasting women were more likely to decrease their overall food intake and to sleep less during Ramadan, as compared to non-fasting pregnant Muslims.

The most common reason for fasting was that the study participants felt strong enough to fast on the days that they fasted (62%). At the same time, sickness was most often reported as reason against fasting (66%) (Appendix Table 1). The most common sources consulted for information on Ramadan during pregnancy were family (85%), friends/acquaintances (33%) and the internet (15%).

**Table 1:** Maternal characteristics, behavior during Ramadan and offspring neonatal health by maternal fasting status during pregnancy in a sample of 1370 singleton mother-child pairs in Kaduna, Nigeria

Variable	Fasting (*N* = 1158)	Non-Fasting (*N* = 212)	Total *N* with information	*P* value (Pearson X²)
	Sample Share	Sample Share		
**Adaptations to Ramadan**				
Number of days fasted				
*3–9*	5.61%		1370	
*10–19*	13.47%			
*20–30*	80.92%			
Decreased food intake during Ramadan	39.17%	28.02%	1333	0.002
Slept less than usually during Ramadan	13.95%	8.10%	1364	0.020
Consumed less of fatty foods during Ramadan	40.37%	N/A		
Consumed less fruits during Ramadan	24.70%	N/A		
Drank less during Ramadan	23.16%	N/A		
**Maternal Characteristics**				0.368^a^
Highest educational level				
*No formal education*	0.33%	0%		
*Quranic school*	5.43%	7.09%	1061	
*Primary school*	12.93%	17.02%		
*Secondary school*	53.04%	48.23%		
*Tertiary*	28.26%	27.66%		
Homemaker (not employed)	45.46%	43.60%	1368	0.617
Lived in Kaduna for less than 5 years	8.55%	10.38%	1370	0.388
Informed herself about Ramadan during pregnancy	91.27%	96.68%	1368	0.007
Other household members fasted	97.67%	96.21%	1368	0.217
Primipara	32.93%	43.87%	1369	0.002
**Infant characteristics**				
Gestational duration at birth (weeks) ^b^	38.99 (2.05)	38.8 2 (2.17)	1364	0.266 ^c^
Term birth (≥ 37 & ≤42 gest weeks)	88.45%	86.32%	1364	0.377
Female baby	52.99%	52.11%	1061	0.845
Birth weight ^b^	3005.96 (472.78)	3206.74 (514.82)	1059	0.001 ^c^

Note that some participants lacked complete information on certain characteristics. The column “Total N with information” indicates the total number of observations in the sample which have information on the respective variable

a, Mann Whitney U test

b, Mean (SD)

c, t-test

### Ramadan fasting and birth weight

**Fig. 2:**
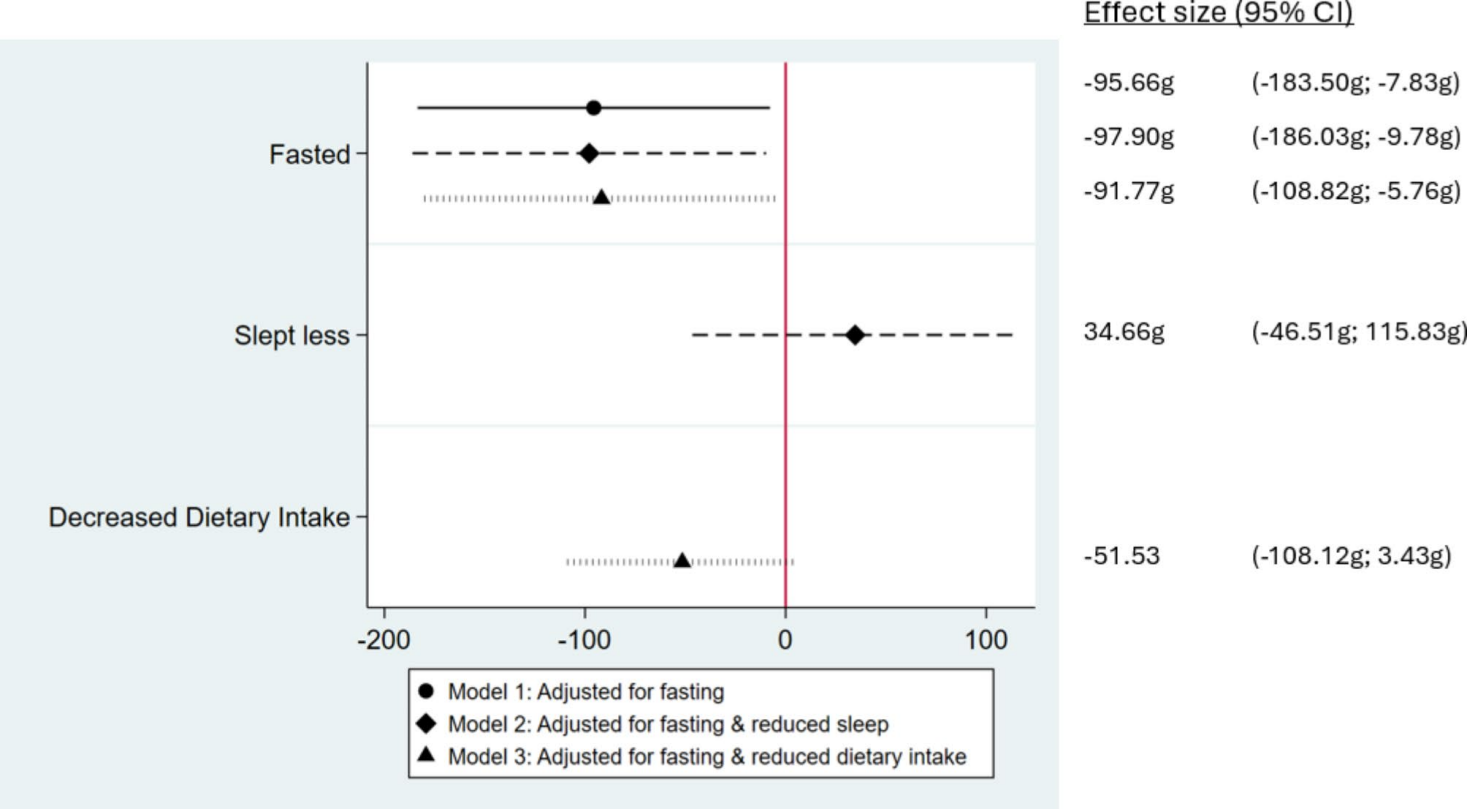
Associations between fasting, sleep and dietary intake during pregnancy with offspring birth weight

**Fig. 3:**
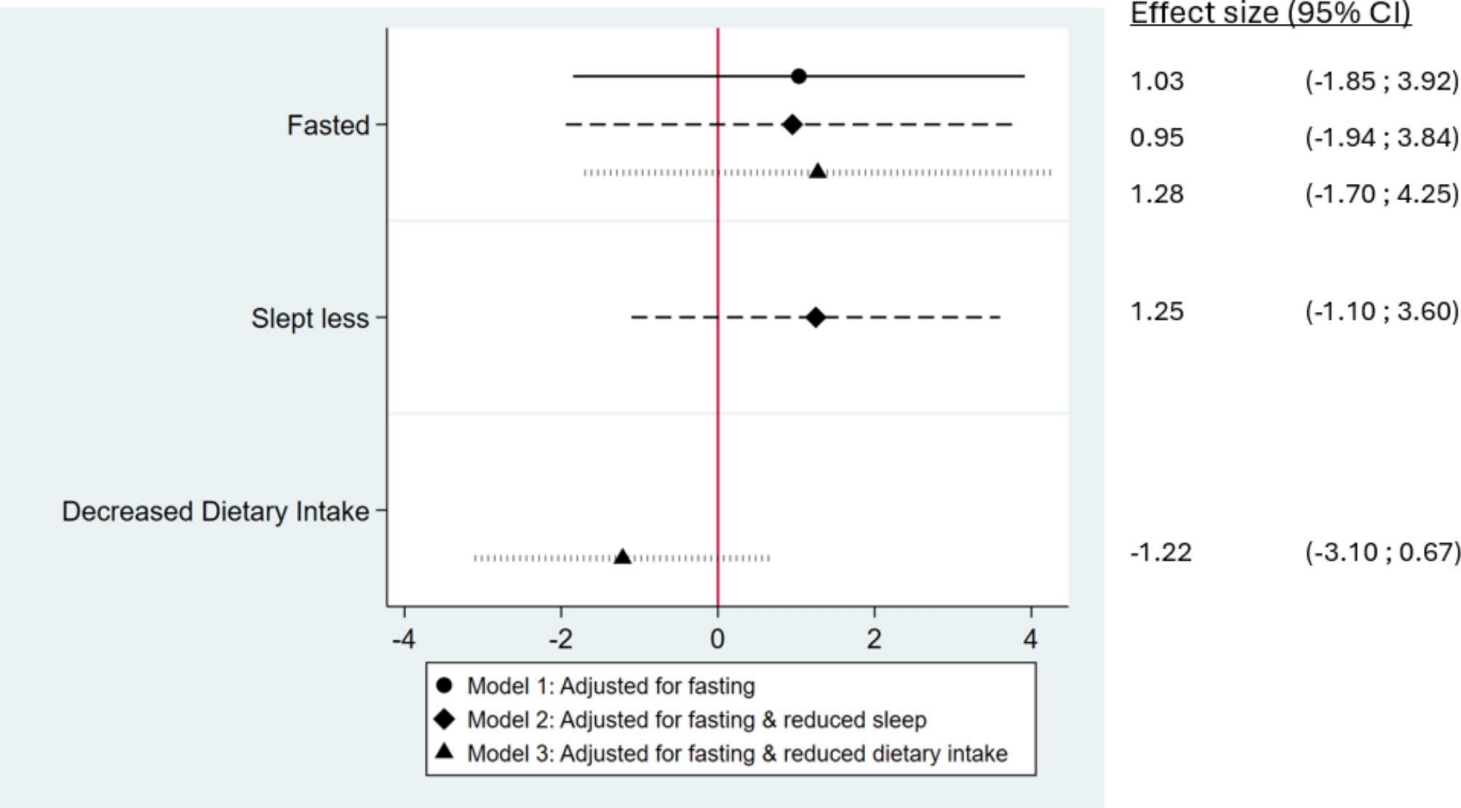
Associations between fasting, sleep and dietary intake during pregnancy with offspring gestational duration

Participants who fasted had infants with lower birth weights compared to non-fasting participants (-90.38 g, 95% CI: -173.64 g; -6.12 g) as shown in Fig. 2, Model 1. There were no independent associations detected between reduced sleep and decreased food intake and birth weight in models further adjusting for reduced sleep and decreased food intake, and adding sleep reduction and food intake reduction to the model did not alter the magnitude of the fasting-birthweight association (Fig. 2, Models 2 and 3). Fasting was not associated with gestational duration in any of these specifications (Fig. 3).

This figure shows the results of three adjusted regressions. All regressions were adjusted for the covariates specified in the manuscript. In addition to fasting, Model 2 adjusted for reduced sleep during Ramadan, and Model 3 for reduced dietary intake during Ramadan. The reference group are non-fasting women. Birth weight was measured in grams.

This figure shows the results of three adjusted regressions. All regressions were adjusted for the covariates specified in the manuscript except for gestational age. In addition to fasting, Model 2 adjusted for reduced sleep during Ramadan, and Model 3 for reduced dietary intake during Ramadan. The reference group are non-fasting women. Gestational duration was measured in days.

To assess the role of behavioral adaptations to Ramadan beyond fasting, we interacted sleep reduction and decreased dietary/fluid intake with fasting, which essentially splits the effect of fasting into those fasting women who reported less sleep/dietary intake during Ramadan vs. those who did not. Statistically significant negative effects of fasting on birth weight were consistently concentrated among participants who reduced their dietary or fluid intake during Ramadan (Table 2). At the same time, fasting only had significant effects on birth weight among fasting women who did not sleep less during Ramadan.

**Table 2:** Associations between fasting during pregnancy and birth weight, by dietary intake during non-fasting hours and sleep during Ramadan

	(1) General food intake	(2) Sweets	(3) Fatty foods	(4) Fruit	(5) Fluids	(6) Sleep
Fasting * Reduced intake during Ramadan	-124.05** [-222.74; -25.39]	-122.24** [-221.64; -22.84]	-111.36** [210.31; 12.41]	-138.03** [-245.76; -30.31]	-142.78** [-253.56; -32.00]	-43.38 [-160.13; 73.36]
Fasting * No reduced intake during Ramadan	-64.32 [-158.30; 29.65]	-63.54 [-157.19; 30.11]	-72.78 [-166.76; 21.20]	-72.25 [-164.62; 20.12]	-72.16 [-164.33; 20.01]	-96.78** [-188.93; -4.63]
N	914	909	910	914	914	916

The table displays the results of six adjusted regressions (columns [1] to [6]). In each regression, the fasting variable is interacted with the dietary intake/behavior specified in the column heading. Birth weight is measured in grams

The reference group are offspring to study participants who did not fast during pregnancy

95% confidence intervals in brackets^**^p < 0.05, ^***^p < 0.01

### Sensitivity analyses

Effect patterns on birth weight were similar when extending the sample to include pre- and post-term births (Appendix Table 2). Adjusting for maternal age (Appendix Table 3) led to a considerably lower sample size. Effect patterns on dietary intake remained stable, but the effects of fasting no longer differed by adaptations of the sleep rhythm. The results were stable against the exclusion of single covariates (Appendix Table 4).

## Discussion

This study found a high adherence to Ramadan fasting among pregnant women in Kaduna, Northern Nigeria. We showed that fasting was associated with reduced birth weight, while gestational age at birth remained unaffected. Notably, the significant reduction in birth weight was observed only among fasting women who also reduced their dietary or fluid intake during Ramadan.

This study contributes to the limited evidence on the role of behavioral adjustments – besides fasting - during Ramadan for pregnancy outcomes. Consistent with a study in Indonesia, where a reduction in the total energy, macronutrient as well as water intake was documented among fasting pregnant women [40], we observed a stronger reduction in dietary intake among fasting women. Similar patterns were also documented in Germany [27]. Conversely, in rural Bangladesh, dietary diversity of pregnant Muslims increased during Ramadan, particularly on the days pregnant women fasted [11]. While it appears likely that these differences could be explained by factors such as access to food, culture, or urbanicity, they highlight the need for future research to shed more light on these variations. For example, in settings with significant food scarcity, increased dietary diversity and overall food intake may be observed among certain population groups due to traditional meals at the breaking of the fast, and the provision of free food to the poor.

The association between fasting and reduced birth weight was significant only for women who decreased their intake of food and fluids across all studied categories. It is important to note that adaptations during Ramadan across these categories were highly correlated, making it impossible to identify specific food items or food groups as decisive factors. Nevertheless, our findings make a timely contribution to the literature on Ramadan and neonatal health outcomes. The evidence on Ramadan during pregnancy and birth weight has to date remained inconclusive, with some studies documenting decreased birth weight and others reporting no association [16, 41]. While differences in study design and target populations have mainly been suggested as potential reasons for these inconsistent findings so far, our study underscores the potential importance of Ramadan-related behaviors beyond the fasting decision. This is the second study worldwide to link fasting, dietary intake and sleep patterns. In both German and Nigerian settings – despite their differences – the negative effects of fasting were concentrated among women who decreased their dietary intake during Ramadan. Since dietary practices outside of fasting hours are relatively easily modifiable, to the extent that access to food is secure, further research into the role of diet during Ramadan among pregnant fasting Muslims is warranted.

Sleep disruption is another potential pathway through which Ramadan fasting may affect fetal development. Fasting persons eat exclusively at night. Food preparation and consumption are shifted to nighttime hours, often resulting in reduced sleep [25, 26]. While a study from Germany found that the negative effects of fasting were concentrated among women who also experienced reduced sleep, we in contrast find negative effects among women who did not reduce their sleep during Ramadan. However, this effect disappeared after adjusting for maternal age, so that we recommend a cautious interpretation. Future studies using assessments of sleep quality, in addition to sleep quantity, might yield important insights.

Our study is not without limitations. While it is one of the largest survey-based studies on Ramadan during pregnancy worldwide, we faced constraints in the availability of some covariates and outcomes. A significant challenge was the inconsistent documentation of birth dates and the comparatively large number of children for whom birth outcomes were not recorded. One main contributing factor to this issue is that some children are not brought to the immunization unit until more than a week after delivery. Nurses are instructed not to document birth weight in such circumstances. Similar observations have been noted in previous research conducted in the setting [42]. Importantly, however, our results were consistent across different sample specifications and covariate combinations. We recommend that future studies place greater emphasis on the thorough institutional documentation of all outcomes and covariates. For instance, it would be beneficial for subsequent research to systematically collect data on maternal weight prior to pregnancy and maternal age at the time of birth. This approach would help compensate for gaps in hospital records.

Additionally, it is important to note that the representativeness of our sample is confined to pregnant Muslim women who visited our study sites with their offspring. Consequently, our sample does not include home births that were not presented for vaccination shortly after birth. Unfortunately, we were unable to investigate specific biological mechanisms through which fasting might impact intrauterine fetal growth and development. For example, lifestyle changes associated with Ramadan might slow placental growth due to dietary alterations beyond daytime fasting [43]. While the assessment of placental health was beyond the scope of this study, we plan to address this important issue in future research. Additionally, our assessment of maternal nutrition was based on self-reported data. To explore the role of specific micronutrients, future research could include detailed nutritional assessments during the fasting period. Finally, this study relies on a structured questionnaire with mostly closed questions. Given the cultural and religious significance of Ramadan, more qualitative research on the experience of Ramadan during pregnancy for Muslim women as well as their prenatal caregivers could provide valuable insights.

## Conclusions

Ramadan during pregnancy is of high public health relevance in Kaduna (Northern Nigeria), where there is a high population of Muslims. Our results underline the potential importance of dietary intake during the non-fasting hours of Ramadan, confirming recent findings on Ramadan during pregnancy in other parts of the world. This study also highlighted the necessity for additional research across different settings, including an assessment of the role of specific macro- and micronutrients. Ramadan traditions vary considerably across and within countries, so that additional evidence is pivotal for designing comprehensive guidelines that can assist pregnant Muslims and their healthcare providers during the pregnancy and family planning phase globally.

## Electronic supplementary material

Below is the link to the electronic supplementary material.

Supplementary Material 1

Supplementary Material 2

## Data Availability

This research relies on a relatively small dataset, and the data we collected contains details about the participants that could potentially identify them. We need specific sensitive data, such as birth dates, to compute essential variables for our analysis, including the overlap of Ramadan with pregnancy. Additionally, the risk of identifying individuals is heightened because the city of Kaduna sees a limited number of births each day, and our focus on Muslim births makes it even more likely that individual births are recognized. Thus, releasing this data to the public could compromise participant privacy. However, researchers who fulfill the requirements for accessing confidential data can obtain access through the ethics committees of Kaduna State Ministry of Health and Human Services, and the Johannes Gutenberg University Mainz, at the Gutenberg School of Management and Economics.
