# Supplementary material for: Ramadan during pregnancy and the role of dietary intake for neonatal health in Kaduna, Northwestern Nigeria: a cross-sectional study
Source: BMC Pregnancy Childbirth. 2025 Jan 23;25:59. doi: 10.1186/s12884-025-07158-2 (PMC11758726; doi:10.1186/s12884-025-07158-2)
Supplement: Supplementary file 1 — Supplementary Material 1 [file 12884_2025_7158_MOESM1_ESM.pdf]

Questionnaire "Pregnancy during Ramadan"

Note: Everything that is written in red should not be read out loud! (Support for the interviewer)

Introduction

Short introduction: Hello, my name is__________. We, at the Kaduna Infant Development (KID) Birth Cohort Study, are conducting a survey study among Muslim women about the topic pregnancy and Ramadan. I would appreciate it, if I could askyou some questions. The interview will last around 5-10 minutes.

1. Are you:

   O Muslim

   O Other religion

2. We are interviewing Muslim women who are either pregnant or have recently delivered about how they experienced Ramadan. May I ask you some questions about this?

   O Yes

   O No, I would not like to participate in this study. → **End of questionnaire**

For the interviewer:

If the woman is interested in participating in the survey study:

- *Ask, if the interview can be conducted right then and there or if she prefers going somewhere else*
- *Introduce the project using the info-sheet, especially explain the confidentiality clause*
- *Mention that all Muslim women are of interest to us, whether or not they fasted during Ramadan*
- *Fill out the informed consent sheet and have the woman sign it (incl. their agreement that the survey data will be linked with the data of the birth registry/ data of the obstetric department)*

Name	____________			
Birth date (dd.mm.yyyy)	____________			
Birth number(n)	____________			
Date (dd.mm.yyyy)	____________			
Hospital	__YDMH ________**O** Others (Name of Hospital or Home)			
Interviewer	☐	Jennifer	☐	Fatima
	☐	Habiba	☐	Others (name)

Before completing the survey, the informed consent form has to be completed and signed!

Pregnancy

3. *In case the child was already born:* When did you give birth to your child? (dd/mm/yyyy)

__________ /__________/ 20__________

4. When is/ was the expected date of delivery? (dd/mm/yyyy)

__________ /__________/ 20__________

Attention: For delivery dates after April 2024, ask whether the woman was pregnant during Ramadan! If no: Continue interview and omit questions that explicitly ask about pregnancy in Ramadan.

5. Is this your first pregnancy or have you been pregnant before?

O This is my first pregnancy.
O I have been pregnant before.
→ How many children do you have (*in case the child was already born*: including your newborn)?

____

Of these____________pairs of twins.

Ramadan - general questions

6. Did you fast for one or more days during the Ramadan of **2023**?

O Yes → ***continue with question 7 (on page 3)***
O No, I did not fast → ***continue with question 15 (on page 5)***

Ramadan – questions for women who fasted

I would like to start out by asking you some questions about the days you fasted.

7. How many days did you fast during the Ramadan 2023?

O On all days (29 days) → ***continue with question 9***
O On most days (20-28 days) → ***starting here continue with question 8***
O On about half of the days (10-19 days)
O On some days (3-9 days)
O On few days (1-2 days)

In case an explanation or the exact number of days fasted were offered, please note down:

8. Are you planning to make up the days you did not fast?

O I already made up these days
O Yes, I am planning to make up these days at the following point in time:

O No, because:

9. On the days you fasted, did you fast normally – in other words, did you give up food and drink during the day?

O Yes
O No

10. Did you abstain from anything else during the day, for example medicine?

Medicine: O Yes O No
O Other:

The next few questions are about your diet on the days on which you fasted.

11. Compared to the month before Ramadan, did you:

**eat**:	O more	O less	or	O the same amount	
**drink**:	O more	O less	or	O the same amount	
**eat**:	O more	O less	or	O the same amount	**of fruit and vegetables**
**eat**:	O more	O less	or	O the same amount	**of sweets**
**eat**:	O more	O less	or	O the same amount	**of greasy/ fatty food**

O Further changes to my diet:

12. In comparison to your diet during Ramadan when you are not pregnant, did you change your diet during this Ramadan because you were pregnant? Please consider both **what** and **how much** you ate and drank.

O Yes, I changed my eating habits in the following way:

O No, I did not change my eating and drinking habits because I was pregnant

13. What did you eat during the breaking of the fast?

14. Why did you fast during this Ramadan?

Ramadan - all

15. Did other members of your household fast?

O No

O Yes, the following people fasted:

O Husband O Parents O Parents-in-law O Children O Others:

***In case respondent fasted:* I would now like to ask you several questions about those days during Ramadan 2022, on which you did NOT fast.**

16. We are interested in whether or not your diet also changed on those days on which you did **not** fast – because, for example, you still celebrated the breaking of the fast with your family. On the days on which you did not fast, compared to the month directly before Ramadan, did you eat:

O more	O less	or	O the same amount	**in the evening/ at night**
O more	O less	or	O the same amount	**during the day**
O more	O less	or	O the same amount	**sweets and candy**

O Further changes:

O This question does not apply because I fasted on all days of Ramadan.

17. Why did you <u>not fast / not fast on all days</u> during this Ramadan?

O This question does not apply because I fasted on all days of Ramadan.

18. Do you normally fast when you are not pregnant?

O No → ***continue with question 20***

O Yes, I normally fast: → ***continue with question 19***

O On all days (29/30 days)

O On most days (20-28/29 days)

O On about half of the days (10-19 days)

O On some days (3-9 days)

O On few days (1-2 days)

In case an explanation or the exact number of days fasted were offered, please note down:

19. In case you are not able to fast on all days, do you make these days up at a later point in time?

    O Yes
    O No
    If further comments/ explanations are given:

20. What effect do you think fasting during Ramadan has on the health of the unborn child?

    O No effect
    O Negative effect
    O Positive effect
    O Depends on the health of the mother
    O No (direct) answer

    If further comments are made in regards to the type of impact, please note down:

21. What effect do you think fasting during Ramadan has on the health of a pregnant woman?

    O No effect
    O Negative effect
    O Positive effect
    O No (direct) answer

    If further comments are made in regards to the type of impact, please note down:

22. What does your partner think about fasting during pregnancy?

    O He believes that pregnant women should fast
    O He believes that pregnant women should **not** fast
    O He has no opinion
    O I do not know
    O I do not have a partner
    O Other:

23. Were you already aware of your pregnancy during Ramadan 2023?

O Yes → ***continue with question 25***
O No → ***continue with question 24***
O I found out during Ramadan that I was pregnant → ***continue with question 24***

24. If you had known about your pregnancy, would you have changed your fasting behavior? If so, how? (*Or* if you became aware of your pregnancy during Ramadan, did you change your behavior?)

    O Yes, I would have/ did change:

    O No

25. Did you inform yourself about fasting during pregnancy? *(please mark all that apply)*

    O Yes, using the following sources of information:
    0 internet
    0 family
    0 friends/ acquaintances
    0 magazines/ books
    0 Other:

    O No, I did not inform myself

26. Did you discuss your fasting plans with your midwife and/ or gynecologist?

O Yes → ***continue with question 27***
O No → ***continue with question 28***

27. What advice did your midwife / your gynecologist give you?

28. Did you suffer from morning sickness during this Ramadan?

    O No, never
    O Yes, sometimes
    O Yes, often

29. Did you get up earlier during Ramadan 2022 than you normally do in comparison to the month before Ramadan?

    O Yes
    O Sometimes
    O No
    If further comments/ explanations are given:

30. Did you go to bed later during Ramadan 2023 than you normally do in comparison to the month before Ramadan?

    O Yes
    O Sometimes
    O No
    If further comments/ explanations are given:

31. Did you sleep more during the day during Ramadan 2023 than you normally do in comparison to the month before Ramadan?

    O Yes
    O Sometimes
    O No
    If further comments/ explanations are given:

32. Did you fast during past pregnancies?

    O Yes
    O No
    O Not applicable, since this is the first pregnancy
    O Not applicable, since past pregnancy was not during Ramadan
    O I do not know / I do not remember

    In case other things are mentioned, please note down:

33. How tall are you?_______________________m

34. a. How much did you weigh before your pregnancy?__________________kg

    b. How much weight did you gain during your pregnancy?____________________kg

    (Alternatively –how much do you weigh today?_______________kg)

KID Study

Personal information

Finally, I would like to ask you for some personal information.

35. What is your date of birth? (dd/mm/yyyy) ________/________/________

36. What is your ethnic group?

37. *If you were not born in Kaduna:* how long have you been living in Kaduna?
years

38. Is your partner Muslim?
O Yes O No O I do not have a partner

39. What is your partner's ethnic group?

40. What is your mother's ethnic group?

41. What is your father's ethnic group?

42. Which of the following situations was most applicable to you before this pregnancy (before the beginning of your parental leave)? *(please mark all answers that apply)*
O Working full time
O Working part time
O Unemployed
O Student
O Not working (such as housewife)

43. What is your highest educational degree?
O No degree / elementary school
O Secondary school
O Completed vocational training
O Technical college
O University degree (bachelor/ master)

Alternatively: Until what age did you go to school? years

44. Do you wear a veiling in every day life?
O No
O Yes, I wear:
0 A hijab (Face free, hair/ears/neck covered)
0 A burqa (Body and face covered, net in front of eyes)
0 A niqab (Face completely covered, narrow slit for the eyes)
0 A chador (dark cloth, hair and body covered, face free)

End of questionnaire

The survey is now completed. Thank you very much for your participation! If you have any other comments or questions, feel free to ask them now.

If the respondent has further comments, please note them down here:

KID Study

To be completed by the interviewer:

1. Was the interviewee wearing a headscarf during the interview?
   0 No
   0 A hijab (Face free, hair/ears/neck covered)
   0 A burqa (Body and face covered, net in front of eyes)
   0 A niqab (Face completely covered, narrow slit for the eyes)
   0 A chador (dark cloth, hair and body covered, face free)

2. Was the interviewee alone during the interview?
   0 Alone (no accompanying person)
   0 Accompanied by her partner
   0 Other:
      0 Child (this does not mean the newborn baby
      0 Man
      0 Woman
   0 Interview in shared room (room neighbor in room)

3. Did the interview take place during an extended inpatient stay before delivery?
   O Yes
   O No

Further comments/ remarks/ suggestions from the interviewer:
