# Supplementary material for: Ramadan during pregnancy and the role of dietary intake for neonatal health in Kaduna, Northwestern Nigeria: a cross-sectional study
Source: BMC Pregnancy Childbirth. 2025 Jan 23;25:59. doi: 10.1186/s12884-025-07158-2 (PMC11758726; doi:10.1186/s12884-025-07158-2)
Supplement: Supplementary file 2 — Supplementary Material 2 [file 12884_2025_7158_MOESM2_ESM.docx]

**Appendix Table 1. Individual motivations for and against Ramadan fasting during pregnancy in a sample of 1814 women whose pregnancy overlapped with Ramadan 2023 in Kaduna, Nigeria**

|  | |  |
| --- | --- | --- |
| **Panel A. Reasons for Fasting during Pregnancy**  **“On the days that you fasted: Why did you fast?”** | Share all fasting women | Share women fasting >20 days |
| Feeling strong/capable | 62% | 67% |
| Religion | 22% | 23% |
| Wanted to try it out | 7% | 1% |
| No appetite anyhow | 4% | 5% |
| Felt like fasting / fasting as normal thing to do | 4% | 4% |
| Does not want to make up for fast later | 3% | 3% |
|  |  |  |
| **Panel B. Reasons against Fasting during Pregnancy “On the days that you did not fast: Why not?”** | Share all participants with at least 1 non-fasted day | Share non-fasting women |
| Sickness | 66% | 54% |
| Hunger | 14% | 38% |
| Pregnancy | 13% | 19% |
| Need to rest | 9% | <1% |
| Feeling weak | 7% | 5% |

The table summarizes the reasons for fasting among fasting women (Panel A) and the reasons against fasting for days on which women did not fast (Panel B). The table is based on an open question to which women could provide multiple answers. Trained interviewers noted down all answers given. Answers were categorized independently by two research assistants. In case of discrepancies, a third researcher was consulted.

**Appendix Table 2. Associations between fasting during pregnancy and birth weight, by dietary intake during non-fasting hours and sleep during Ramadan. Sensitivity check: Full sample including births outside of the term window.**

|  | (1)  General food intake | (2)  Sweets | (3)  Fatty foods | (4)  Fruit | (5)  Fluids |  | (6)  Sleep |
| --- | --- | --- | --- | --- | --- | --- | --- |
| Fasting * Reduced intake during Ramadan | -104.64**  [-197.49; -11.78] | -107.78**  [-200.78;-14.77] | -99.27**  [-191.97; -6.56] | -129.85**  [-232.67,-27.02] | -129.31**  [-234.52 ; -24.10] |  | -31.93  [-140.97; 77.12] |
| Fasting * No reduced intake during Ramadan | -71.96  [-159.88; 15.96] | -67.16  [-155.12 ; 20.80] | -74.78  [-162.97; 13.42] | -69.77  [-156.02; 16.47] | -71.45  [-157.55; 14.66] |  | -94.22**  [-180.38; -8.06] |
| N | 1,045 | 1040 | 1041 | 1045 | 1045 |  | 1048 |
|  |  |  |  |  |  |  |  |

The table displays the results of six adjusted regressions (columns (1) to (6)). In each regression, the fasting variable is interacted with the dietary intake/behavior specified in the column heading. Birth weight is measured in grams.
The reference group are offspring to study participants who did not fast during pregnancy.

95% confidence intervals in brackets^**^ *p* < 0.05, ^***^ *p* < 0.01

**Appendix Table 3. Associations between fasting during pregnancy and birth weight, by dietary intake during non-fasting hours and sleep during Ramadan. Sensitivity check: Regressions adjusted for maternal age at birth.**

|  | (1)  General food intake | (2)  Sweets | (3)  Fatty foods | (4)  Fruit | (5)  Fluids | (6)  Sleep |
| --- | --- | --- | --- | --- | --- | --- |
| Fasting * Reduced intake during Ramadan | -135.80** [-262.68; -8.93] | -136.99** [-265.69; -8.31] | -117.69** [-246.04; 10.66] | -168.22** [-308.58; -27.83] | -158.64** [-301.64; -15.63] | -38.51 [-198.09; 121.06] |
| Fasting * No reduced intake during Ramadan | -26.51 [-147.02; 94.01] | -21.85 [-142.16; 98.45] | -37.30 [-157.52; 82.92] | -36.47 [-155.30; 82.37] | -39.91 [-158.69; 78.86] | -69.14 [-186.19; 47.90] |
| N | 577 | 572 | 573 | 577 | 577 | 579 |
|  |  |  |  |  |  |  |

The table displays the results of six adjusted regressions (columns (1) to (6)). In each regression, the fasting variable is interacted with the dietary intake/behavior specified in the column heading. Birth weight is measured in grams.
The reference group are offspring to study participants who did not fast during pregnancy.

95% confidence intervals in brackets^**^ *p* < 0.05, ^***^ *p* < 0.01

**Appendix Table 4. Associations between fasting during pregnancy and birth weight, by dietary intake during non-fasting hours and sleep during Ramadan. Sensitivity check: Test of the stability of results against the exclusion of single covariates**

|  | (1) | (2) | (3) | (4) | (5) | (6) | (7) | (8) | (9) |
| --- | --- | --- | --- | --- | --- | --- | --- | --- | --- |
| Panel A. General Food Intake | | | | | | | | | |
| Fasting * No reduced general food intake | -64.32 | -63.97 | -71.083 | -69.72 | -64.91 | -64.24 | -71.94 | -59.58 | -65.41 |
|  | [-158.30; 29.65] | [-157.17; 29.23] | [-161.671,19.505] | [-163.41; 23.97] | [-158.57; 28.74] | [-158.02; 29.55] | [-165.30; 21.41] | [-152.95; 33.79] | [-159.54; 28.71] |
|  |  |  |  |  |  |  |  |  |  |
| Fasting * Reduced general food intake | -124.05** | -120.28** | -126.77** | -123.78** | -123.26** | -124.11** | -128.76** | -117.72** | -122.18** |
|  | [-222.74; -25.39] | [--218.16; -22.40] | [-225.88; -27.67] | [-222.04; -25.51] | [-221.76; -24.76] | [--222.77; -25.44] | [-227.55; -29.98] | [-215.66; -19.79] | [-221.14; -23.22] |
| *N* | 914 | 917 | 914 | 917 | 914 | 914 | 914 | 918 | 914 |
| Panel B. Sweets | | | | | | | | | |
| Fasting * No reduced sweets intake | -63.54 | -62.95 | -65.57 | -68.45 | -63.99 | -63.41 | -71.18 | -58.54 | -65.40 |
|  | [-157.19; 30.11] | [-155.88; 29.96] | [-159.56; 28.42] | [-161.83; 24.93] | [-157.46; 29.47] | [-156.83; 30.01] | [-164.38; 22.03] | [-151.58; 34.49] | [-159.19; 28.40] |
|  |  |  |  |  |  |  |  |  |  |
| Fasting * Reduced sweets intake | -122.24** | -118.96** | -125.71** | -121.72** | -121.32** | -122.34** | -127.90** | -116.50** | -119.06** |
|  | [-221.64; -22.84] | [-217.55; -20.38] | [-225.86; -25.56] | [-220.79; -22.65] | [-220.49; -22.15] | [-221.66; 23.01] | [-227.24; -28.56] | [-215.16; -17.84] | [-218.77; -19.35] |
| *N* | 909 | 912 | 909 | 912 | 909 | 909 | 909 | 913 | 909 |

| Panel C. Fluids | | | | | | | | | |
| --- | --- | --- | --- | --- | --- | --- | --- | --- | --- |
| Fasting * No reduced fluids intake | -72.16 | -70.47 | -72.76 | -75.01 | -71.94 | -72.10 | -78.80 | -67.31 | -71.39 |
|  | [-164.33; 20.01] | [-161.85; 20.91] | [-165.35; 19.84] | [-166.86; 16.84] | [-163.88; 19.98] | [-164.08; 19.89] | [-170.58; 12.97] | [-158.77; 24.16] | [-163.77; 20.98] |
| Fasting * Reduced fluids intake | -142.78** | -141.25** | -150.02*** | -144.52*** | -143.32** | -142.87** | -147.45*** | -135.47** | -144.46** |
|  | [-253.56; -32.00] | [-251.35; -31.17] | [-261.29; -38.75] | [-254.88; -34.17] | [-253.99; -32.65] | [-253.49; -32.25] | [-258.28; -36.63] | [-245.36; -25.59] | [-255.34; -33.58] |
| *N* | 914 | 917 | 914 | 917 | 914 | 914 | 914 | 918 | 914 |
| Panel D. Fatty Foods | | | | | | | | | |
| Fasting * No reduced fatty foods intake | -72.78 | -72.54 | -73.64 | -78.36 | -73.11 | -72.58 | -79.82 | -67.68 | -74.68 |
|  | [-166.76; 21.20] | [-165.78; 20.71] | [-168.01; 20.73] | [-172.03; 15.31] | [-166.87; 20.66] | [-166.34; 21.18] | [-173.27; 13.63] | [-161.01; 25.64] | [-168.78; 19.42] |
|  |  |  |  |  |  |  |  |  |  |
| Fasting * Reduced fatty foods intake | -111.36** | -107.52** | -114.94** | -110.19** | -110.67** | -111.50** | -116.82** | -105.27** | -108.35** |
|  | [210.31; 12.41] | [-205.59; -9.46] | [-214.53; -15.35] | [-208.63; -11.74] | [-209.43; -11.92] | [-210.36; -12.63] | [-215.77; -17.88] | [-203.48; -7.06] | [-207.59; -9.11] |
| *N* | 910 | 913 | 910 | 913 | 910 | 910 | 910 | 914 | 910 |

| Panel E. Fruit Intake | | | | | | | | | |
| --- | --- | --- | --- | --- | --- | --- | --- | --- | --- |
| Fasting * No reduced fruit intake | -72.25 | -70.66 | -73.42 | -75.56 | -71.95 | -72.18 | -78.26 | -67.23 | -72.03 |
|  | [-164.62; 20.12] | [-162.24; 20.91] | [-166.16; 19.32] | [-67.57; 16.46] | [-164.09; 20.18] | [-164.36; 19.99] | [-170.22; 13.71] | [-158.98; 24.50] | [-164.60; 20.54] |
|  |  |  |  |  |  |  |  |  |  |
| Fasting * Reduced fruit intake | -138.03** | -136.09** | -143.40*** | -139.21** | -138.72** | -138.13** | -143.54*** | -131.60** | -138.03** |
|  | [-245.76; -30.31] | [-243.05; -29.14} | [-251.80; -35.00] | [-246.88; -31.54] | [-246.34; 31.10] | [-245.69; -30.56] | [-251.28; -35.80] | [-238.67; -24.53] | [-246.02; -30.03] |
| *N* | 914 | 917 | 914 | 917 | 914 | 914 | 914 | 918 | 914 |
| Panel F. Sleep | | | | | | | | | |
| Fasting * no reduced sleep | -96.78** [-188.93; -4.63] | -94.89** [-186.16; -3.63} | -97.96** [-190.44; -5.48] | -98.89** [-190.63; -7.16] | -96.72** [-188.66; -4.77] | -96.74** [-188.79; -4.70] | -102.43** [-194.22; -10.65] | -94.11** [-184.09; -4.12] | -96.70** [-189.05; -4.35] |
| Fasting * reduced sleep | -43.38 [-160.13; 73.36] | -42.15 [-158.70; 74.40] | -44.61 [-161.77; 72.56] | -50.36 [-167.18; 66.46] | -43.46 [-159.95; 73.03] | -43.06 [-159.63; 73.40] | -50.20 [-166.73; 66.32] | -39.02 [-154.05; 76.01] | -42.92 [-159.76; 73.92] |
| N | 916 | 919 | 916 | 919 | 916 | 916 | 916 | 920 | 916 |
| **Included Covariates** | | | | | | | | | |
| *Baby sex* | X |  | X | X | X | X | X | X | X |
| *Pregnancy trimester of Ramadan overlap* | X | X |  | X | X | X | X | X | X |
| *Maternal education* | X | X | X |  | X | X | X | X | X |
| *Gestational age at birth* | X | X | X | X |  | X | X | X | X |
| *New to Kaduna* | X | X | X | X | X |  | X | X | X |
| *Primipara* | X | X | X | X | X | X |  | X | X |
| *Mother was aware of pregnancy during Ramadan* | X | X | X | X | X | X | X |  | X |
| *Employment status* | X | X | X | X | X | X | X | X |  |

This table shows the results of nine adjusted regression analyses per behavioral change during Ramadan, organized in panels per behavioral change. While Column (1) reports the baseline result (see Table 2), in each of the following columns, single covariates are taken out of the regression, with the respective covariate sets being specified at the bottom of the table. Birth weight is measured in grams.

The reference group are offspring to study participants who did not fast during pregnancy.

95% confidence intervals in brackets

^**^ *p* < 0.05, ^***^ *p* < 0.01
